# Involvement of Notch Signaling in Wound Healing

**DOI:** 10.1371/journal.pone.0001167

**Published:** 2007-11-14

**Authors:** Srinivasulu Chigurupati, Thiruma V. Arumugam, Tae Gen Son, Justin D. Lathia, Shafaq Jameel, Mohamed R. Mughal, Sung-Chun Tang, Dong-Gyu Jo, Simonetta Camandola, Marialuisa Giunta, Irina Rakova, Nazli McDonnell, Lucio Miele, Mark P. Mattson, Suresh Poosala

**Affiliations:** 1 Laboratory of Neurosciences, National Institute on Aging Intramural Research Program, Baltimore, Maryland, United States of America; 2 Laboratory of Clinical Investigation, National Institute on Aging Intramural Research Program, Baltimore, Maryland, United States of America; 3 Research Resources Branch, National Institute on Aging Intramural Research Program, Baltimore, Maryland, United States of America; 4 Department of Pharmaceutical Sciences, School of Pharmacy, Texas Tech University Health Sciences Center, Amarillo, Texas, United States of America; 5 College of Pharmacy, Sungkyunkwan University, Suwon, Korea; 6 Department of Pathology, Breast Cancer Program, Loyola University Health Science Center, Maywood, Illinois, United States of America; 7 Department of Neuroscience, Johns Hopkins University School of Medicine, Baltimore, Maryland, United States of America; University of Birmingham, United Kingdom

## Abstract

The Notch signaling pathway is critically involved in cell fate decisions during development of many tissues and organs. In the present study we employed *in vivo* and cell culture models to elucidate the role of Notch signaling in wound healing. The healing of full-thickness dermal wounds was significantly delayed in Notch antisense transgenic mice and in normal mice treated with γ-secretase inhibitors that block proteolytic cleavage and activation of Notch. In contrast, mice treated with a Notch ligand Jagged peptide showed significantly enhanced wound healing compared to controls. Activation or inhibition of Notch signaling altered the behaviors of cultured vascular endothelial cells, keratinocytes and fibroblasts in a scratch wound healing model in ways consistent with roles for Notch signaling in wound healing functions all three cell types. These results suggest that Notch signaling plays important roles in wound healing and tissue repair, and that targeting the Notch pathway might provide a novel strategy for treatment of wounds and for modulation of angiogenesis in other pathological conditions.

## Introduction

Notch-1 (Notch) is a cell surface receptor that regulates cell fate decisions during development; depending on the cell type and context, Notch signaling induces differentiation or maintains cells in an undifferentiated proliferating state [1]–[4]. Binding of ligands of the Delta or Jagged families results in proteolytic cleavages of Notch, first in an extracellular domain and then in the transmembrane domain. The latter cleavage is accomplished by the γ-secretase enzyme complex resulting in the release of a Notch intracellular domain (NICD) that translocates to the nucleus where it regulates transcription [5]. Growing evidence implicates Notch signaling in the regulation of tissue homeostasis in adults. For example, Notch regulates lymphocyte expansion and immune function [6], synaptic plasticity [7] and neural cell responses to injury [8] in the adult rodent brain. Notch signaling is also involved in angiogenesis, the formation of new blood vessels [9]–[11]. Mutations of Notch receptors and ligands in mice lead to abnormalities in many tissues, including the vascular system. It was shown that mice lacking Notch [10] or the Notch ligand Jagged-1 [11] die during embryonic development as a result of vascular plexus remodeling defects. Similarly, haploinsufficiency of Jagged-1 in humans results in Alagille syndrome, characterized among other things by congenital vascular abnormalities that are a significant cause of mortality [12]. In addition, Notch signaling regulates endothelial cell proliferation and migration during angiogenesis in normal tissues and tumors [13]–[16].

Wound healing involves an initial inflammatory response and subsequent changes in keratinocytes, fibroblasts and vascular endothelial cells that close the wound and regenerate the skin tissue [17]. Although it is not known if Notch plays a role in wound healing, recent studies demonstrated the expression of Notch and the Notch ligands Jagged-1 and Jagged-2 and Notch in vascular endothelial cells in situ [18]. In addition, Notch signaling has been reported to affect angiogenesis [19], [20]. Notch has also been shown to affect the behaviors of keratinocytes, fibroblasts and platelets [21]–[25], additional cell types that play important roles in wound healing.

In the present study we employed Notch antisense transgenic mice (NAS), γ-secretase enzyme inhibitors and the Notch ligand Jagged-1 to elucidate the role of Notch signaling in wound healing. Our data demonstrate a pivotal role for Notch signaling in wound healing in vivo, as well as direct positive effects on endothelial, keratinocyte and fibroblast cells. These findings reveal Notch signaling as a novel therapeutic target for the treatment of wounds.

## Results

### Wound healing is impaired in Notch antisense transgenic mice and normal mice treated with a γ-secretase inhibitor and enhanced in mice treated with Jagged-1 peptide

**Figure 1:**
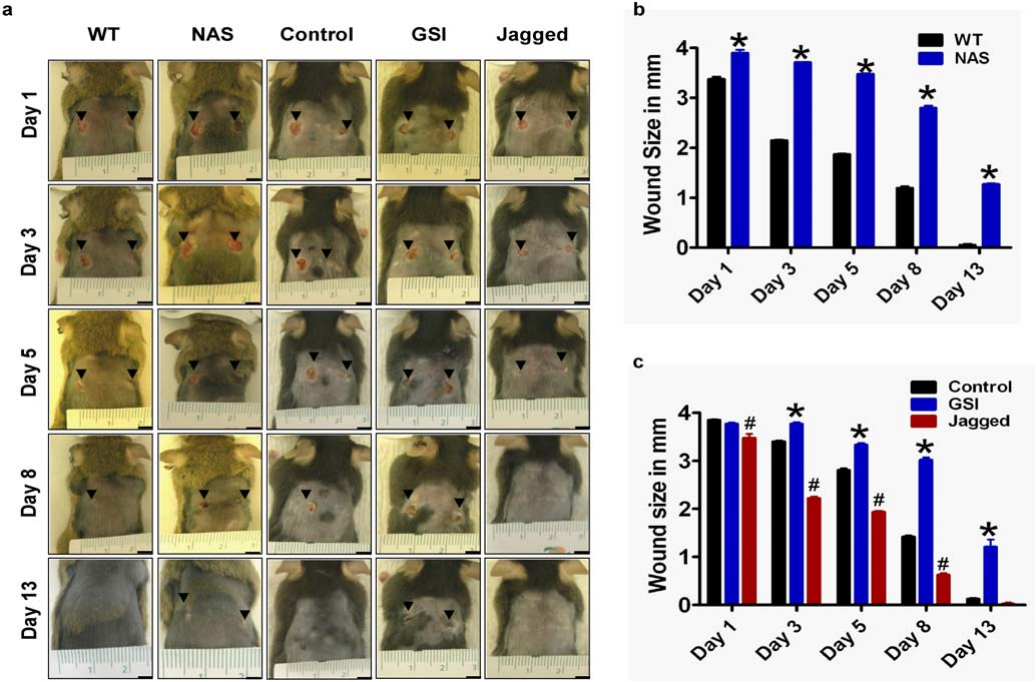
Genetic and pharmacological inhibition of Notch impairs wound healing. Two full-thickness dermal wounds were induced in NAS mice and nontransgenic mice, vehicle-treated control mice, g-secretase inhibitor (GSI)-treated (100 µM DAPT) mice and 15 µM mouse Jagged peptide-treated mice. a. Images of a representative mouse from each group taken on post-injury days 1, 3, 5, 8 and 13 are shown. b. Wound sizes at the indicated time points in NAS and wild-type control mice (WT). c. Wound sizes at the indicated time points in control, GSI-treated and Jagged peptide-treated mice. Values are the mean and SEM (n = 6 mice per group). *p<0.001, #p<0.01 compared to the control value. Scale bar = 4 mm.

We first investigated the role of Notch in the wound healing process by comparing the rate of dermal wound healing in mice with reduced levels of Notch (NAS mice) and nontransgenic control mice. In nontransgenic control mice, 4 mm full-thickness dermal wounds healed rapidly with the lesions being reduced by 50% within 5 days, and were completely healed within 13 days (Fig. 1a, b). In contrast, healing was delayed in NAS mice, with the lesion size being decreased by only 15% at 5 days, and not being completely healed at 13 days. We next treated the wounds of normal mice with the γ-secretase inhibitor DAPT to inhibit the activation of Notch within cells involved in wound healing. Compared to vehicle-treated control mice, those treated with DAPT exhibited a significant delay in wound healing (Fig. 1a, c). To further confirm the role of Notch signaling in wound healing, we treated the wounds of normal mice with mouse Jagged-1 peptide to activate the Notch cells within the wound area. Mice treated with Jagged peptide showed significantly enhanced wound healing compared to vehicle-treated control animals (Fig. 1 a, c).

### Reduced Notch signaling perturbs multiple cellular events in the wound healing process

**Figure 2:**
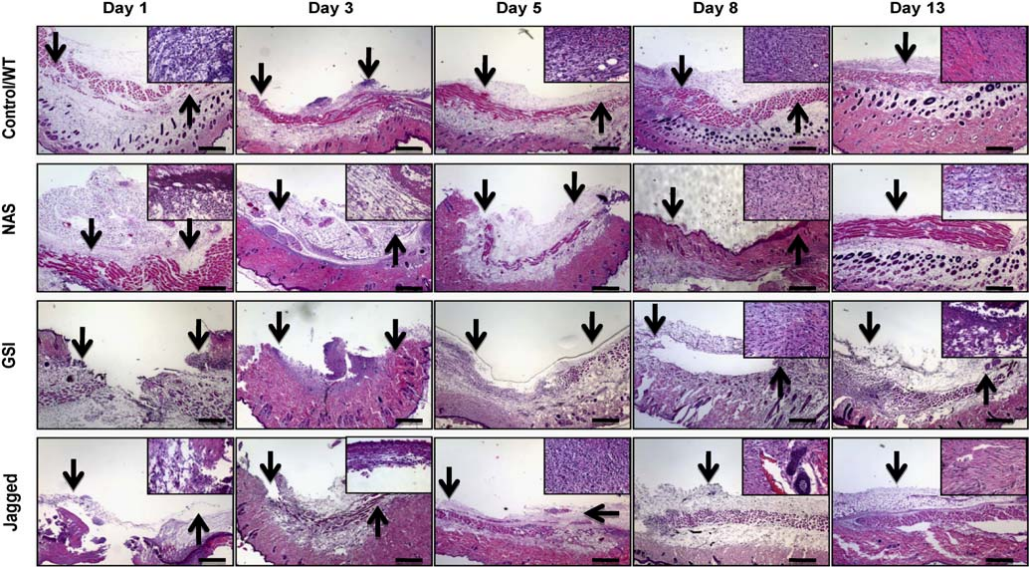
Histological features of wound healing in mice with decreased or increased Notch activity. Images of skin tissue sections stained with hematoxylin and eosin showing histological changes during the wound healing process in non-transgenic control mice, NAS mice, GSI (100 µM DAPT) treated mice and 15 µM Jagged peptide-treated mice at post-injury days 1, 3, 5, 8 and 13. NAS and DAPT-treated mice exhibited delayed wound healing, and Jagged peptide-treated exhibited enhanced wound healing, compared to control mice. See Supplementary histological evaluation (Text S2) for a detailed description of histological changes in the different groups of mice. Scale bar = 0.5 mm

Detailed histopathological analysis of H&E sections showed delayed wound healing in NAS animals at days 1, 3, 5, 8 and 13 post-injury compared to non transgenic control mice (Fig. 2 and see SI histological evaluation). On day 1, nontransgenic control mice exhibited a superficial layer of the wound that was lined by a thin layer of collagen, a damaged keratinocyte layer with a distinct edema underneath with expanded space and fibrin deposits; scattered mononuclear cells and neutrophils were observed within the damaged tissue. On day 1, NAS mice exhibited a larger fibrin-filled spaces and greater numbers of mononuclear cells and neutrophil infiltrates compared to control mice (Fig. 2). On day 3 the edema and fibrinous exudates had resolved substantially in control mice, while mononuclear cells had infiltrated in greater numbers into the interstitium and the dermal layers. NAS mice on day 3 exhibited large numbers of cellular infiltrates and necrotic tissue, and the hypodermis was expanded with edema. On day 5 the wounds in control mice exhibited distinct crusting (eschar), with the skin and wound junction evident beneath the semi-attached crust. The keratinocyte layer was absent at the wound site, but compared to day 3 the edema and fibrin exudates were reduced, the collagen fibers were densely packed and fibroblasts were visible in this matrix. Dense, irregular collagen and minimal edema were observed under higher magnification, and neo-vascularization was evident within the wound site. On day 5, NAS mice had fewer fibers of collagen and fewer migrating fibroblasts in the superficial dermis of the wound site, and there was a thin layer of pre-keratinization (Fig. 2). Little or no neovascularization of the wound site had occurred by post-injury day 5 in NAS mice. On day 8 the wound site of control mice contained large amounts of collagen in the dermis (scar tissue) with few mononuclear cells and numerous distinct blood vessels (Fig. 2). The keratinocyte layer had become well-formed and edema and expanded interstitial spaces were absent. On day 8 the NAS mice exhibited prominent collagen deposition in the superficial dermis, but it was less organized compared to control mice. There were foci of dead cells and necrotic tissue on the wound surface, and mononuclear cell infiltrates were still present intermixed with collagen. Neovascularization was distinctly less than in control mice. At day 13 the skin in the wounds of control mice appeared normal with a distinct orthokeratotic keratinocyte layer, well organized dermis and adnexal structures. There was no indication of inflammation (edema or cell infiltration). Wound healing (eschar shedding and healthy granulation) appeared complete. In contrast, the wounds of NAS mice were not completely healed by day 13 as indicated by the presence of dense collagen deposits in superficial and deeper dermis, and by the presence of mononuclear cells infiltration. Keratinization was in the initial stages. The keratinocyte layer of the wound site was single-layered in comparison to the multilayered keratinocytes in the healthy skin of the same animals (Fig. 2).

To confirm the involvement of Notch signaling in wound healing, we examined H&E stained sections of skin from vehicle- and DAPT and Jagged peptide-treated mice. The histological changes described above for normal wound healing in control mice were delayed in DAPT-treated mice and accelerated in Jagged peptide-treated mice (Fig. 2; and see SI histological evaluation). Collectively, the analyses of wound healing in NAS mice, Jagged and DAPT-treated normal mice suggested that Notch signaling plays important roles in several aspects of cellular responses to dermal wounds including leukocyte infiltration, angiogenesis and keratinocyte migration.

### Notch is activated transiently in the skin in response to wounding

**Figure 3:**
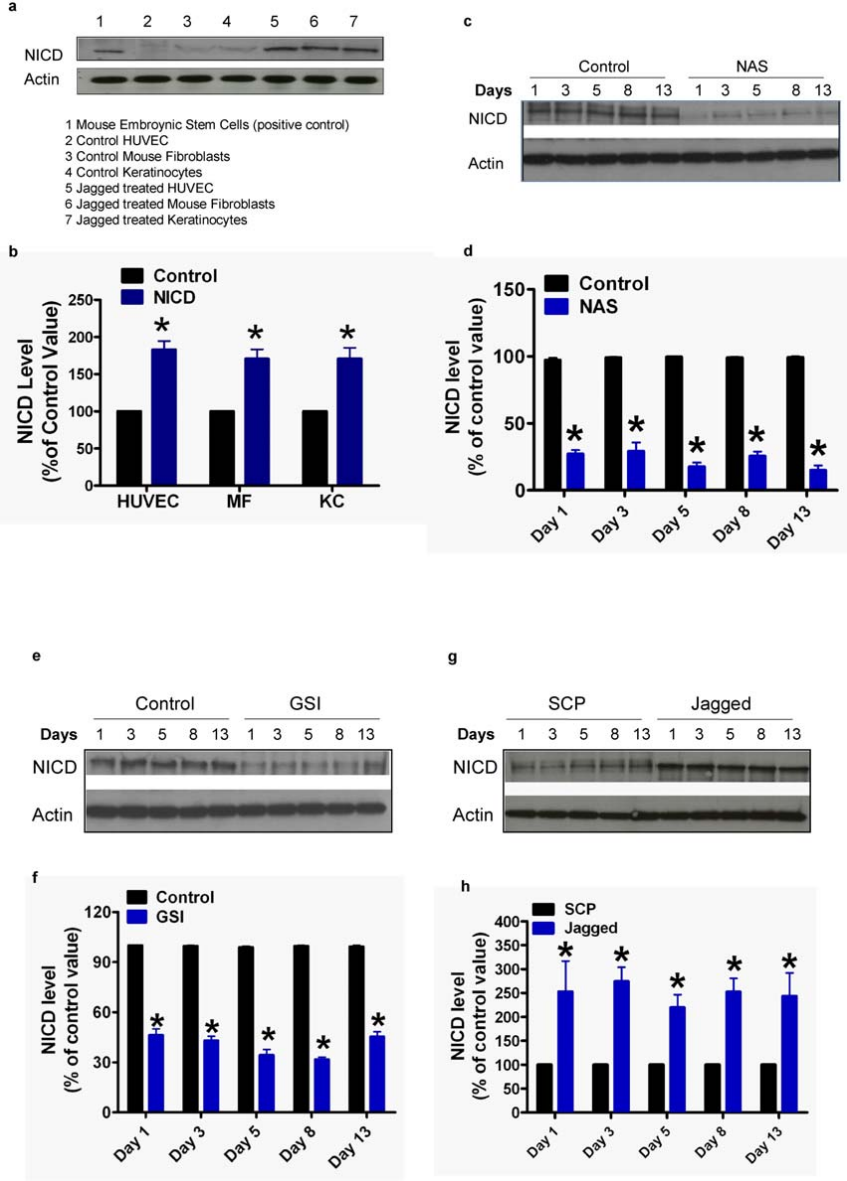
Notch activity in skin cells is decreased in NAS mice and in response to γ-secretase inhibition (10 µM), and is increased in response to Jagged peptide treatment (15 µM). a and b. Representative immunoblot (a), and results of densitometric analysis of blots (b), showing relative levels of NICD (80 kDa) and actin (42 kDa) in lysates (40 µg/lane) of cultured human vascular endothelial cells (HUVEC), mouse keratinocytes (KC) and in mouse fibroblasts (MF) that had been treated for 6 h with vehicle or Jagged peptide. c and d. Representative immunoblot (c), and results of densitometric analysis of blots (d), showing relative levels of NICD in skin tissue samples from NAS and control mice taken at the indicated post-wounding time points. *p<0.01 compared to the control value. e and f. Representative immunoblot (e), and results of densitometric analysis of blots (f), showing relative levels of NICD in skin tissue samples from control and γ-secretase inhibitor (GSI)-treated mice taken at the indicated post-wounding time points. g and h. Representative immunoblot (g), and results of densitometric analysis of blots (h), showing relative levels of NICD in skin tissue samples from control and Jagged peptide-treated mice taken at the indicated post-wounding time points. *p<0.01 compared to the control value (ANOVA p = and Newman-Keuls post hoc tests for pairwise comparisons).

To study the level of Notch signaling activity in response to Jagged peptide treatment in different cell types involved in wound healing, we measured the level of NICD in endothelial cells, keratinocytes and in fibroblasts. We used mouse embryonic stem cells which exhibit high levels of Notch activity as a positive control. Treatment with Jagged peptide significantly increased the level of NICD in all 3 skin cell types (Fig. 3a, b). We next examined the level of NICD in skin samples from the wound sites in NAS and control mice, and DAPT-, Jagged peptide- and vehicle-treated mice. The levels of NICD were significantly lower throughout the 13 day study period in NAS mice compared to nontransgenic controls (Fig. 3c, d). The level of NICD was decreased within 24 h of treatment with DAPT and remained low through treatment day 13 (Fig. 3e, f). The levels of NICD in skin samples from the wound area of Jagged peptide-treated mice were significantly higher throughout the 13 day treatment period compared to vehicle-treated control mice (Fig. 3g, h).

### Notch signaling enhances vascular endothelial cell proliferation, migration and tube formation

Because endothelial cells are critically involved in neovascularization and the wound healing process, we determined the effects of Notch activation and inhibition on the behaviors of cultured human vascular endothelial cells. First, we confirmed that the Notch signaling pathway was functional in endothelial cells, fibroblasts and keratinocytes. This was accomplished by expressing reporter constructs for the Notch target genes CBF-1 and Hes-1 in cultured endothelial cells, keratinocytes and skin fibroblasts. Activation of Notch with Jagged peptide increased CBF-1 and Hes-1 expression in all three cell types (Figure S1).

**Figure 4:**
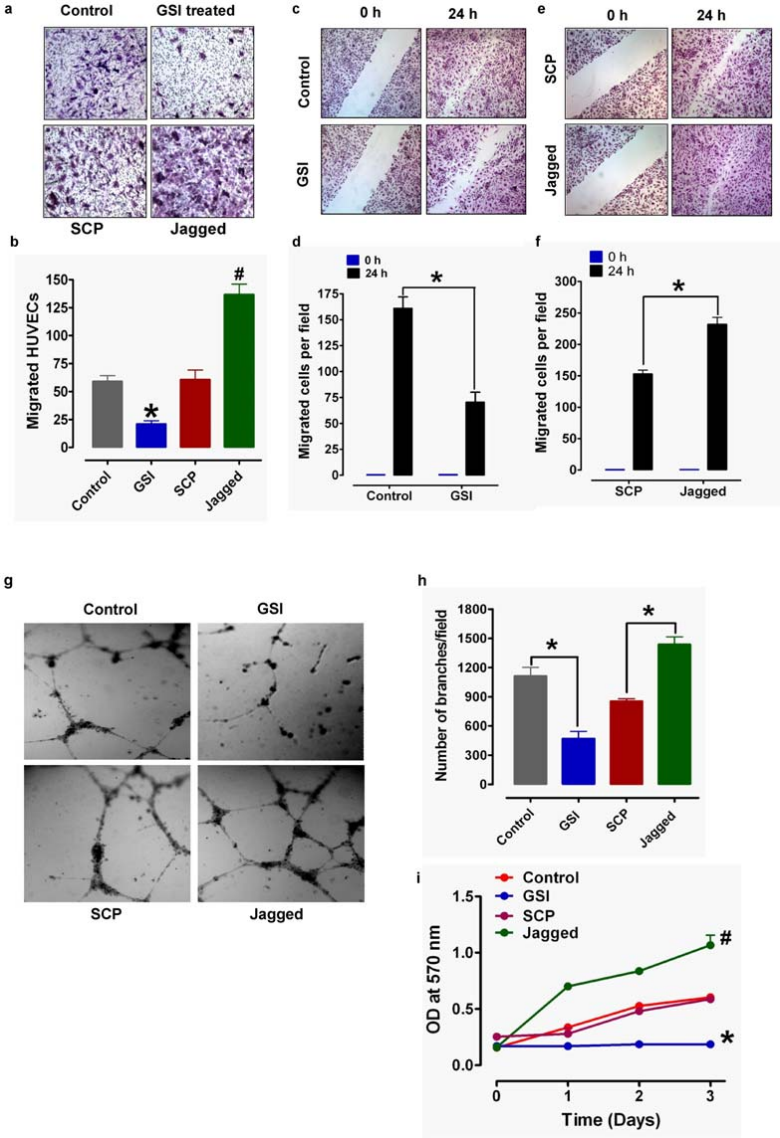
Notch signaling regulates the motility and proliferation of vascular endothelial cells. a and b. Cultured human vascular endothelial cells (HUVEC) were treated with either vehicle (Control), g-secretase inhibitor (GSI; 10 µM DAPT) or Jagged peptide (15 µM) in conditioned medium and plated into chemo-attractant medium consisting regular growth medium with 10% FBS and other growth factors and cell migration was evaluated using a 24 well Transwell chamber assay. Representative images are shown in panel and a and quantitative data are shown in panel b (values are the mean±SEM for cells per 100×field; n = 3-4). *p<0.001 compared to control values. #p<0.001 compared to SCP values. c–f. HUVEC monolayers were mechanically wounded with the sterile tip of a 20–200 µl pipette tip following treatment with vehicle, GSI (10 µM DAPT), scrambled control peptide (SCP; 15 µM) or Jagged peptide (15 µM). Representative images are shown in c and e, and quantitative data on cell migration in d and f. Values are the mean and SEM (n = 3 separate experiments). *p<0.001. g and h. HUVEC were seeded on Matrigel-precoated wells and cultured in the presence of low-serum medium with GSI (10 µM DAPT), SCP (15 µM) or Jagged peptide (15 µM). Tube formation, designated as the number of branch points/100×field) was evaluated 18h after cell plating. Representative images are shown in g and quantitative data in h. Values are the mean and SEM (n = 12–16 cultures). *p<0.001. i. Cultured HUVEC were treated with GSI (10 µM DAPT), scrambled peptide (SCP; 15 µM) or Jagged peptide (15 µM) for the indicated time periods and relative cell numbers were estimated (n = 4–6 experiments). *p<0.001, #p<0.01 compared to the control cultures.

Next, we determined the effects of γ-secretase mediated Notch signaling on the chemotaxis of vascular endothelial cells. Cells in 24-well Transwell chamber assay cultures were treated with vehicle, DAPT, Jagged peptide or SCP, and chemoattractant-induced cell migration was quantified. The numbers of cells that migrated through the transwell pores were significantly lower in DAPT-treated cultures compared to vehicle-treated cultures (Fig. 4a, b). In contrast, Jagged peptide treatment resulted in a significant enhancement of endothelial cell chemotaxis compared to SCP- and vehicle-treated control cultures (Fig. 4a, b). We next examined the endothelial cell migration in response to mechanical wound. Endothelial cells in monolayer culture were subjected to mechanical scratch wound injury in the absence or presence of the γ-secretase inhibitor DAPT (10 µM) to inhibit Notch or a Jagged peptide (15 µM) to activate Notch. The numbers of cells that had migrated into the cell-free wound zone during a 24 hour post-injury period were quantified. Endothelial cell migration was inhibited by more than 50% in cultured treated with DAPT compared to vehicle-treated control cultures (Fig. 4c, d). Treatment of cultures with Jagged peptide resulted in a significant increase in endothelial cell migration compared to control cultures (Fig. 4e, f). To elucidate a role for Notch signaling in angiogenesis, we employed an assay that measures the formation of tubular capillary-like structures by endothelial cells growing in a three-dimensional matrix. Cultures were treated for 18 hours with vehicle, DAPT, Jagged peptide or a control peptide with a scrambled amino acid sequence (SCP), and tube formation was quantified. Both the number of capillary-like structures and their total branch points were significantly lower in DAPT-treated cultures compared to vehicle-treated control cultures (Fig. 4g, h). In contrast, tube formation was significantly greater in cultures treated with Jagged peptide compared to control cultures treated with SCP (Fig. 4g, h). These results suggest a stimulatory effect of Notch signaling on endothelial cell migration, and suggest a role for Notch in angiogenesis during wound healing. Because both proliferation and migration of cells is required for dermal wound healing, we also assessed the effects of Notch inhibition and activation on the proliferation of cultured vascular endothelial cells. γ-secretase inhibitor DAPT completely inhibited cell proliferation during a 3 day treatment period, whereas Jagged peptide significantly enhance the cell proliferation rate compared to cells in control cultures (Fig. 4i).

### Notch signaling affects the migration of keratinocytes and fibroblasts

**Figure 5:**
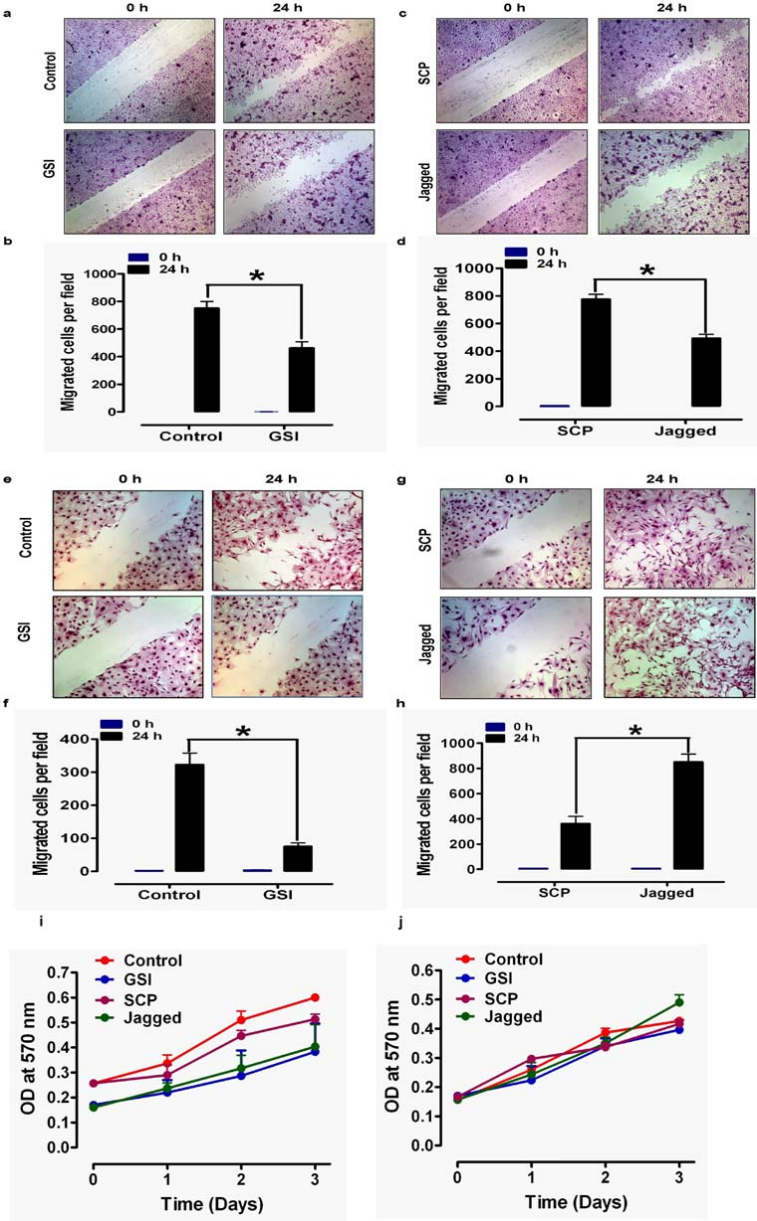
Evidence that Notch signaling regulates the behaviors of keratinocytes and skin fibroblasts. a–h. Monolayers of cultured keratinocytes (a–d) or fibroblasts (e–h) were treated with vehicle (Control), GSI (10 µM DAPT), 15 µM SCP or 15 µM Jagged peptide, and were then subjected to scratch wounding. Eighteen hours after wounding, images of the wound area were acquired and the number of cells per field that had migrated into the cell-free wound zone were determined for each culture. Representative images (a, c, e, g) and quantitative data (b, d, f, h) are shown. Values are the mean and SEM (n = 3 separate experiments). *p<0.001. i and j. Cultured keratinocytes (i) and fibroblasts (j) were treated with vehicle (Control), GSI (10 µM DAPT), 15 µM SCP or 15 µM Jagged peptide for the indicated time periods and relative cell numbers were estimated. Values are the mean and SEM (n = 4–6 experiments).

As both keratinocytes and fibroblasts play major roles in skin wound healing, we next determined the effects DAPT, Jagged peptide and a scrambled control peptide on injury-induced cell migration in monolayer cultures of keratinocytes and fibroblasts. Keratinocyte migration was inhibited by more than 25% in cultures treated with DAPT compared to vehicle-treated control cultures (Fig. 5a, b). Interestingly, Jagged peptide treatment also significantly decreased keratinocyte migration compared to control cultures (Fig. 5c, d). Fibroblast migration was inhibited by more than 65% in culture treated with DAPT (Fig. 5e, f), while Jagged peptide significantly enhanced fibroblast migration (Fig. 5g, h). Neither DAPT nor Jagged peptide significantly affected the proliferation rates of keratinocytes or fibroblasts, although there was a trend towards inhibition of proliferation with both treatments (Fig. 5i, j).

## Discussion

Our findings suggest that Notch signaling affects multiple cell types involved in the skin injury response and dermal repair processes. Wound healing was impaired in NAS mice and in normal mice treated with a γ-secretase inhibitor, and was enhanced in mice treated with the Notch ligand Jagged, demonstrating a requirement for Notch signaling for normal wound healing. The NAS mouse models what partial loss of Notch does in multiple cell types. The antisense cDNA was cloned in the Nhe1 and Xho1 sites of pMAMneo, which was then linearized, SV40 sequences were excised and linear DNA was injected into blastocysts F1 C57bl6/SJL. Two independent founders were derived, both characterized by lighter hair color than wild type littermates (silver and gold). These were bred back into the C57bl6 background for several generations, segregating by PCR done on tail DNA. The antisense is expressed in skin where the only observable phenotype is a discoloration of the hair to a lighter color [26], [27]. Notch signaling is highly dose-dependent. For example, Notch signaling can either maintain the undifferentiated status or promote differentiation of neural stem cells depending on magnitude and duration of activation [28]. Our study design is therefore of therapeutic relevance because it shows what a systemic but partial Notch inhibition does, which is what is likely to happen with GSI treatment in the clinic.

Notch signaling affected several behaviors of vascular endothelial cells that are critical for angiogenesis, a process critical for tissue repair as a wound heals [29]–[32]. Angiogenesis involves endothelial cell migration into the surrounding tissue, cell proliferation, alignment and tube formation, recruitment of parenchymal cells and a return to quiescence [30]–[32]. Activation of Notch with a Jagged peptide enhanced vascular endothelial cell proliferation, migration and tube formation, whereas treatment with a γ-secretase inhibitor suppressed all three behaviors of the endothelial cells. While previous studies indicated a role for Notch in angiogenesis in tumors [33], [34], the present findings are the first to document the involvement of Notch in angiogenesis triggered by dermal injury. Compounds that promote angiogenesis have been reported to enhance wound healing [35]. However, little is known regarding the signaling mechanisms involved in regulating the different stages of an angiogenic response that can be targeted for the development of novel treatments for wounds. Our findings suggest Notch as a novel target for therapeutic intervention to enhance wound healing.

The initial response to wounding is typically the formation of a blood clot, which together with local damaged tissue, releases proinflammatory signals which trigger the infiltration of inflammatory cells including neutrophils and macrophages. These signals and those from the invading inflammatory cells influence both re-epithelialisation and connective tissue contraction of the wound [36]. The inflammatory response may exacerbate tissue damage, but is not essential for skin wound healing [37]. We found that the infiltration of inflammatory cells into the damaged tissue was decreased in NAS mice and by treatment with a γ-secretase inhibitor, suggesting a key role for Notch signaling in the inflammatory cell responses. Indeed, it was recently reported that Notch signaling is pivotal for the recruitment and activation of leukocytes and microglia to the site of tissue injury following an ischemic stroke in mice [8]. Other studies have also suggested roles for Notch signaling in inflammatory processes including the activation of macrophages [38] and suppression of airway inflammation [39]. Enhancement by Notch of inflammatory cell recruitment and activation would be expected to exacerbate the initial tissue damage.

Our findings also suggest roles for γ-secretase and Notch signaling in regulating the behaviors of keratinocytes and fibroblasts, two additional cell types involved in dermal wound healing. Migration of these cells following injury was significantly reduced when γ-secretase was inhibited. Studies of cultured fibroblasts have indeed shown that activation of Notch alters cell migration [40]. We found that activation of Notch with a Jagged peptide enhanced the migration of endothelial cells and fibroblasts, but inhibited the migration of keratinocytes. Although the Notch target genes that regulate cell behaviors during wound healing remain to be established, we did establish that Notch activation results in induction of the Notch target genes CBF-1 and Hes-1 in dermal endothelial cells, keratinocytes and fibroblasts. However, it has been reported that activation of Notch signaling in keratinocytes is sufficient to cause cell cycle withdrawal, through induction of p21^WAF1/Cip1^ and expression of terminal differentiation markers of the intermediate epidermal layers [21]. The latter findings are consistent with our observation that keratinocyte migration was reduced in response to Jagged peptide.

Okuyama and colleagues [41] provided evidence that Notch accounts in part for the high commitment of embryonic keratinocytes to terminal differentiation. Another study showed that soluble form of Jagged1 induced the differentiation of cultured keratinocytes [42]. Levels of Jagged-1 and Notch increased during the differentiation process, and may trigger keratinocyte terminal differentiation and cornification [24]. However, we found that GSI treatment also significantly reduced keratinocyte migration, suggesting that Notch signaling is important for keratinocyte migration and differentiation in wound healing. Our data suggest a beneficial role for Notch signaling in angiogenesis, fibroblast and keratinocyte migration and terminal differentiation in wound healing. It will be of considerable interest to identify the gene targets of Notch in vascular endothelial cells, keratinocytes and skin fibroblasts that promote cell migration and differentiation.

## Materials and Methods

### Mice

The generation and characterization of transgenic mice in which a Notch antisense sequence is expressed under the control of the mouse mammary tumor virus long terminal repeat promoter has been described previously [43]. Notch protein levels are decreased by approximately 50% in various tissues of these mice, including the skin. The genetic background and breeding scheme for these mice are described in the Supplemental Methods online (Text S1).

### Dermal Wounds and Quantification of Healing

Mice were anesthetized using 2 to 2.5% vaporized inhaled isoflurane and the dorsal skin was cleansed with Betadine. Full-thickness dermal wounds were created in the skin on the back of the mouse using a 4-mm biopsy punch (Miltex Instrument, York, PA, USA) and a biotome (Acu Punch, Acuderm Inc., Fort Lauderdale, FL, USA). Mice were treated with vehicle (10 µl of dimethylsulfoxide) or 100 µM of the γ-secretase inhibitor DAPT (*N*-[*N*-(3,5-difluorophenacetyl)-L-alanyl]-*S*-phenylglycine *t*-butyl ester) (Chemicon, Temecula, CA, USA) [44] and 15 µM mouse jagged peptide (Washington Biotechnology, MD, USA) applied directly to the wound site once daily in a blinded manner. Some mice in each group were euthanized on days 1, 3, 5 and 8 post wounding, and skin tissue samples from the wound site were collected from all of the mice for histological and biochemical analyses. Some animals from each genotype/treatment group (n = 6–8) were evaluated daily for 13 days following wounding. Digital photographs of the injury site were taken with a standard-sized dot placed beside the wound; wound size was expressed as the ratio of the wound area to the dot measurement.

### Histology

To assess cellular infiltration into the wounded area, samples from three mice per group were collected on days 1, 3, 5, 8 and 13 during the healing process. To obtain skin samples from the biopsied areas, mice were euthanized with an overdose of sodium pentobarbital and the tissues were subsequently removed by dissection. Formalin-fixed samples were sectioned at 4 µm and stained with hematoxylin and eosin. All the slides were evaluated by a veterinary pathologist in blinded manner.

### Immunoblot analysis

Tissue protein was extracted using T-PER tissue protein extraction buffer with protease inhibitor cocktail (Sigma). Details of the immunoblot protocol are included in the Supplemental Methods online (Text S1).

### Endothelial cell scratch wound healing assay

Human umbilical vein endothelial cells (HUVECs), Human keratinocytes (CRL-2309, ATCC, VA, USA) and mouse fibroblasts were seeded into 60 mm plates and grown to confluency. After 24 hours of serum starvation (DMEM supplemented with 1% FBS), cells were treated with either vehicle or scrambled peptide (controls), DAPT (10 µM) or Jagged-1 peptide (15 µM). The amino sequence of the scrambled control peptide was RCGPDCFDNYGRYKYCF, and the sequence of the Jagged-1 peptide was CDDYYYGFGCNKFGRPRDD. The cell monolayer was then subjected to a mechanical scratch-wound induced using a sterile pipette tip. Cells were then cultured for additional period of 24 hours in a serum-free basal medium in the presence of controls, DAPT or Jagged peptide. Cells were then fixed in a solution of 4% paraformaldehyde in PBS and stained with crystal violet. Cells in the injury area were visualized under phase-contrast optics (10× objective) and the number of cells which had migrated into the initially cell-free scratch are was counted.

### Endothelial tube formation and chemotaxis cell migration assays

These assays were performed using methods similar to those described previously [45]; details are provided in the Supplemental Methods online (Text S1).

### Quantification of cell proliferation

The proliferation of cultured endothelial cells, keratinocytes and fibroblasts was measured using a colorimetric assay with 3-(4, 5-dimethylthiazol-2-yl)-2,5-diphenyl-2*H*-tetrazolium bromide (MTT). Details are provided in the

## Supporting Information

Text S1 (0.02 MB DOC)

Text S2 (0.02 MB DOC)

Figure S1 Jagged peptide increases the levels of the Notch downstream gene target products CBF-1 and Hes-1 in endothelial, keratinocyte and fibroblast cells. HUVEC, keratinocytes and fibroblasts were treated with Jagged peptide or vehicle control and analyzed for CBF-1 and Hes1 using luciferase reporter assays. *p<0.05, **p<0.01 and ***p<0.001 compared to control value.
(0.07 MB TIF)
