# Supplementary material for: Involvement of Notch Signaling in Wound Healing
Source: PLoS One. 2007 Nov 14;2(11):e1167. doi: 10.1371/journal.pone.0001167 (PMC2048753; doi:10.1371/journal.pone.0001167)
Supplement: Text S1 — (0.02 MB DOC) [file pone.0001167.s001.doc]

Supplemental Methods online Methods
Mice. The genetic background of the founders was (C57BL/6 x SJL) F1, and the mice were then backcrossed for four generations with C57BL/6 mice. Hemizygous transgenic mice were then bred to each other, selecting homozygous transgenic and negative mice until two syngeneic strains derived from the same original litter were obtained, one homozygous NAS and one nontransgenic (control). Transgene integration and expression were confirmed by PCR and RT-PCR, respectively. All procedures described in this study were approved by the National Institute on Aging Animal Care and Use Committee and complied with NIH guidelines.  Immunoblot analysis. Protein concentration was determined using a BCA protein assay kit (Pierce, Rockford, IL, USA). Proteins (50 ìg) were separated by SDS-PAGE (8-12%) and transferred to a nitrocellulose membrane. The membrane was incubated in 5% nonfat milk for 1 hour at room temperature, followed by an overnight incubation at 4 °C with antibodies against NICD (Cell Signaling Technology, Danvers, MA, USA) or â-actin (Sigma, St. Louis, MO, USA). Membranes were then washed and incubated with secondary antibodies for 1 hour at room temperature. Protein bands were visualized using a chemiluminescence detection kit (Amersham Biosciences, Piscataway, NJ, USA). 
Endothelial cell scratch wound healing assay. Human umbilical vein endothelial cells (HUVECs), Human keratinocytes (CRL-2309, ATCC, VA, USA) and mouse fibroblasts (see Supplementary methods) were seeded into 60 mm plates and grown to confluency. After 24 hours of serum starvation (DMEM supplemented with 1% FBS), cells were treated with either vehicle or scrambled peptide (controls), DAPT (10 µM) or Jagged peptide (15 µM). The cell monolayer was then subjected to a mechanical scratch-wound induced using a sterile pipette tip. Cells were then cultured for additional period of 24 hours in a serum-free basal medium in the presence of controls, DAPT or Jagged peptide. Cells were then fixed in a solution of 4% paraformaldehyde in PBS and stained with crystal violet. Cells in the injury area were visualized under phase-contrast optics (10X objective) and the number of cells which had migrated into the initially cell-free scratch are was counted.  
In vitro endothelial tube formation and cell migration assays. HUVECs (1 x 10^3^ cells/well) were dispensed to Matrigel-coated 8-well chamber slides (Lab-Tek, Nalge Nunc International, Rochester, NY, USA) in 125 µl of EGM-2 containing either vehicle or scrambled peptide (controls), DAPT (10 µM) or Jagged peptide (15 µM) and incubated for 18 hours. The cells were then visualized by microscopy and tube formation was scored as described previously [1]. Cell migration analysis was performed using Transwell membrane filters (Corning Costar) containing a polycarbonate filter with 8 µm pores. The bottom chamber was filled with complete growth medium containing chemoattractant growth factors.  Cells (5 × 10^4^ in 100 µl) were seeded into each transwell with EGM containing 0.2% FCS with controls (vehicle or scrambled peptide), DAPT (10 µM) or Jagged peptide (15 µM) and allowed to migrate for 6 hours. At the end of the incubation, non-migrated cells remaining in the transwell insert were removed. The migrated cells (on the outer bottom of the transwell) were fixed with methanol and stained with hematoxylin and eosin, and the stained cells were counted in 5 or more random 100X fields. Each experiment was performed in triplicate, and the experiment was repeated twice. Growth correction was not applied because no increase in the cell number was observed during the incubation period of 6 hours. 
Quantification of cell proliferation.  Cells (1 x 10^4^) were incubated with either vehicle or scrambled peptide (controls), DAPT (10 µM) or Jagged peptide (15 µM) for 24, 48 and 72 hours. Then 10 µl of MTT solution (R&D Systems Inc. Minneapolis, MN) was added to each well and the cells were incubated for a further 4 hours at 37oC. After the cells were washed 3 times with PBS (pH 7.4), the insoluble formazon product was dissolved by incubation with 100 ìl detergent for 2 hours. The absorbance of each well was measured on an enzyme-linked immunosorbent assay (ELISA) micro-plate reader at 570 nm. Each experiment was performed in quadruplicate. The proliferation rate was calculated as follows: (absorbance experimental/Absorbance control-1) ×100.  
Primary culture of fibroblasts: Fibroblast harvesting was done using dermal explants isolate from the skin of young adult mice.   Dermal tissue specimens were cut into 5 mm pieces. These fragments were placed on the surface of 100 mm Petri dishes for 40 minutes to allow adherence of the tissue to the culture surface.  Ten millilitersof DMEM with 20% fetal bovine serum, penicillin (100 UI/ml) and streptomycin (100 ìg/ml) (pH 7.6), at 37°C, was gently added to the culture dishes. poured in the dish. Cultures were maintained in a humidified incubator at 37°C in a 5% CO2/95% air atmosphere.  After 57 days, fibroblasts were harvested by trypsinization and subcultured into the culture dishes used for cell migration or proliferation assays.   
Nucleofection and Luciferase assay:  HUVEC, keratinocytes and mouse skin fibroblasts were dissociated in 0.25% trypsin EDTA and counted.  Cell pellets were resuspended in 100 µl of Nucleofector buffer (cell line nucleofector kit V) (Amaxa Biosciences, Gaithersburg, MD, USA) with 3 µg of plasmid DNA (CBF-1 & HES-1) and 0.5 µg of control vector pRL-TK Vector (Promega) containing renilla luciferase as a transfection efficiency control. Nucleofection was performed using the Nucleofector I device (Amaxa Biosciences). Immediately after nucleofection, cells were plated onto 12-well plates. Culture medium was changed 12 h after nucleofection to remove dead cells, and cells were pretreated with DAPT or Jagged peptide for 24 h. Luciferase activity was quantified in a luminometer using the dual-luciferase-reporter system (Promega). For this, the culture medium was removed and 150 ml of 1x PLB (Passive Lysis Buffer, Promega) was added to each well. Firefly luciferase activity was measured after the addition of a mix containing beetle luciferase substrate (LAR I: Luciferase Assay Reagent I, Promega) to the cell lysate. Then renilla luciferase activity was measured by adding a mix containing coelenterazine (Stop & Glo: Renilla Assay Reagent, Promega). All experiments were performed in triplicate. 
References 
1.	Chigurupati S, Kulkarni T, Thomas S, Shah G (2005) Calcitonin stimulates multiple stages of angiogenesis by directly acting on endothelial cells. Cancer Res 65: 8519-8529. 
