# Supplementary material for: Involvement of Notch Signaling in Wound Healing
Source: PLoS One. 2007 Nov 14;2(11):e1167. doi: 10.1371/journal.pone.0001167 (PMC2048753; doi:10.1371/journal.pone.0001167)
Supplement: Text S2 — (0.02 MB DOC) [file pone.0001167.s002.doc]

Supplemental Information: Histological Evaluation 
Non-Transgenic Control Mice 
Day 1 4X: Edema is evident with expanded space and fibrin in the deep dermis and the adjacent wound site. Numerous polymorphonuclear and mononuclear cells are present within the tissue at the damage site.  The skin edges are healthy and the wound extends to the surface of the deep dermis.  The superficial layer of the wound is lined by a thin crusting.   40X (Insert): Edema and fibrin strands are evident, and accumulations of mononuclear and polymorphonuclear cells are present. 
Day 3 4X: The wound edge with adjacent healthy skin is distinct.  Edema and fibrous appearance have resolved substantially. The interstitial layer and the dermis has greater numbers of mononuclear cells. 
Day 5 4X: There is a partially attached eschar on the surface of the wound with a distinct demarcation between the healthy and wound edges.  The edema and fibrin has substantially disappeared in contrast to day 3.  The collagen fibers are disoriented in the midst of numerous fibroblasts, and new small capillaries have begun to appear. 40X (Insert): Increased numbers of fibroblasts and neo-vascularization are evident. 
Day 8 4X: There is distinct constriction of the wound due to scar tissue formation, typified by numerous new blood vessels, fibroblasts and partly organized collagen.  The wound shows a distinctly constricted appearance, an indication of wound closure. 40X (Insert): More organized connective tissue and neo-vascularization are evident. 
Day 13 4X: The wound site dermis has somewhat less dense collagen compared to the adjacent healthy dermis.  The collagen fibers are thick with a tendency to form irregular bundles.  Nevertheless, the wound has a constricted and the dermis and looks very well organized in comparison to day 8.  There is also a distinct keratinocyte layer on the surface. 40X (Insert): There is a distinct demarcation of healthy and wound site within the dermis.  The staining of the healthy side is more distinctly pink due to well organized bundles of collagen. 

Notch Antisense (NAS) transgenic mice 
Day 1 4X: The wound edges are clear and the wound extended into the deeper dermis.  The clot and crusting on the top is evident. There is extensive edema, fibrin deposits, and polymorphonuclear infiltrates. 
40X (Insert): Extensive crusting and polymorphonuclear cells are evident. 
Day 3 4X: The debridement of the wound is still incomplete.  There is distinct crusting, polymorphonuclear cell infiltration and expanded fibrin and edema.  No evidence of healing as the initial debridement and inflammatory phase is still the most predominate feature. 40X: Distinct edema and expansion of the interstitial space is evident along with mononuclear cells. 
Day 5 4X: The wound has fewer collagen fibers and fewer migrating fibroblasts.  Little or no neo-vascularization is present at the site. 
Day 8 4X: Fibroblasts and disoriented collagen fibers are present along with necrotic cell debris and monocuclear cell infiltrates.  Little or no neo-vascularization is seen. 40X: Disoriented collagen fibers along with fibroblasts and very few new capillaries that are sparsely distributed are evident. 
Day 13 4X: The wound appears partly healed with broken and disorganized collagen fibers. Neo-vascularization and scar tissue formation is still in progress.  The wound healing appears to have been extended due to lack of initial debridement and enhanced inflammation.   40X: Evidence of neo-vascularization indicative of initiation of wound healing, but the collagen density and orientation is not complete. 

DAPT-treated mice 
Day 1 4X: The wound has expanded edema and fibrin strands.  The wound has extended deeper and the deeper dermis seems to have detached from the healthy dermis.  Infiltration of polymorphonuclear cells and monocytes is evident in the wound and the adjacent sites. 
Day 3 4X: The wound has extended deeper with excess fibrin. The muscle layer is also exposed. The crush artifact on the surface is evident.  The wound exhibits excessive infiltrates of mononuclear and polymorphonuclear cells, the presence of necrotic tissue debris, and a hypodermis expanded by edema.   
Day 5 4X: The connective tissue is distinctly loose and the remnants of eschar on the top are evident. There is a greater than normal amount of fibrin, but the mononuclear cells appear reduced in numbers. 
Day 8 4X: The fibrin strands along with expanded interstitial space and polymorphonuclear cells are still evident. 40X: The expanded interstitial space with several cell types including fibroblasts is apparent.  Also, adjacent to this, there is a zone of neo-vascularization. 
Day 13 4X: The wound has new scar tissue with fewer leukocytes in the deeper layers of the dermis.  Collagen fibers in the scar tissue are disoriented with increased numbers of fibroblasts. The keratinocyte layer on the top of the wound is also evident. 40X: Mononuclear cells are still present along with irregular collagen fibers.  Neovascularization is not fully accomplished. 

Jagged-treated mice 
Day 1 4X: The wound has extensive crusting and the edges are clearly demarcated with one edge separated (artifact). There is a preponderance of fibrin, mononuclear and polymorphonuclear cells.  The interstitial space of the deep dermis is edematous and has necrotic cell debris. The wound extended to the surface of the deep dermis. 
Day 3 4X: Extensive crusting is still prominent due to the incomplete detachment of the eschar.  However, the amount of fibrin and cell infiltration is modest.  The dermis has more collagen fibers, fibroblasts and some capillaries, and is less edematous than in control mice.  The keratinocyte layer has distinct desmosomal junctions. 40X (Insert): Fibroblasts and new blood vessels are evident. A new keratinocyte layer with distinct desmosomal junctions is evident. 
Day 5 4X: There is distinct demarcation of the wound edges and fibrin is absent in the wound site. There is marked proliferation of several cell types giving the tissue a densely populated appearance. The amount of collagen is relatively low compared with the enhanced numbers of cells. There are numerous fibroblasts and fewer mononuclear cells with poorly organized collagen fibers. 40X (Insert): Fibroblasts, disorganized collagen fibers and mononuclear cells are evident. 
Day 8 4X: The wound edges are clearly demarcated, with the healthy edge having distinctly mature collagen along with associated adnexal structures and a thin hyperkeratotic keratinocyte layer. The wound has a low amount of mature collagen.  The wound has a constricted appearance due to the contracted scar tissue and the deeper layer has 
infiltration of adipocytes as well. The wound appears closed and healed, but is distinct 
from adjacent undamaged skin. 
40X (Insert): Distinct demarcation between the healthy and wound site collagen.
Numerous fibroblasts, small capillaries and disorganized collagen fibers are evident. 

Day 13
4X: The wound is completely healed with a keratinocyte layer, fibroblasts and 
endothelial cells forming an integral part of the repaired tissue.  However, the collagen at 
the wound site is not too well organized in comparison to the uninjured skin.    
